# Eosinophilic Ascites: A Rare Case Report With Diagnostic and Therapeutic Challenges

**DOI:** 10.7759/cureus.11362

**Published:** 2020-11-06

**Authors:** Sujata Devi, Nilanjan Kar, Debananda Sahoo, Anupam Dey, Dhriti Sundar Das

**Affiliations:** 1 Internal Medicine, All India Institute of Medical Sciences, Bhubaneswar, IND; 2 General Medicine, All India Institute of Medical Sciences, Bhubaneswar, IND

**Keywords:** eosinophilic ascites, eosinophilic gastrointestinal disease, eosinophilic esophagitis, eosinophilic gastroenteritis, eosinophilic colitis

## Abstract

Eosinophilic ascites is a manifestation of serosal eosinophilic gastrointestinal disease. We present a 44-year-old male with low serum ascites albumin gradient with high eosinophil count and contrast-enhanced computed tomography of the abdomen showing circumferential wall thickening of the esophagus, mid to distal ileal loops, and ascending colon. The patient was managed with tablet prednisolone 20 mg twice daily for two weeks, then gradual tapering over one month. The patient responded to treatment. Awareness of the condition, timely diagnosis, and early treatment carries excellent responses.

## Introduction

Eosinophilic ascites is a manifestation of serosal eosinophilic gastrointestinal disease (EGID). It is the least common variety of EGID and commonly associated with peripheral eosinophilia. It is a diagnostic challenge since clinical scenario, imaging studies & biopsy are not always conclusive, and hence it warrants special consideration after ruling out close differentials. Treatment with corticosteroids, however, shows an excellent response with fewer chances of recurrence or steroid resistance.

## Case presentation

**Figure 1:**
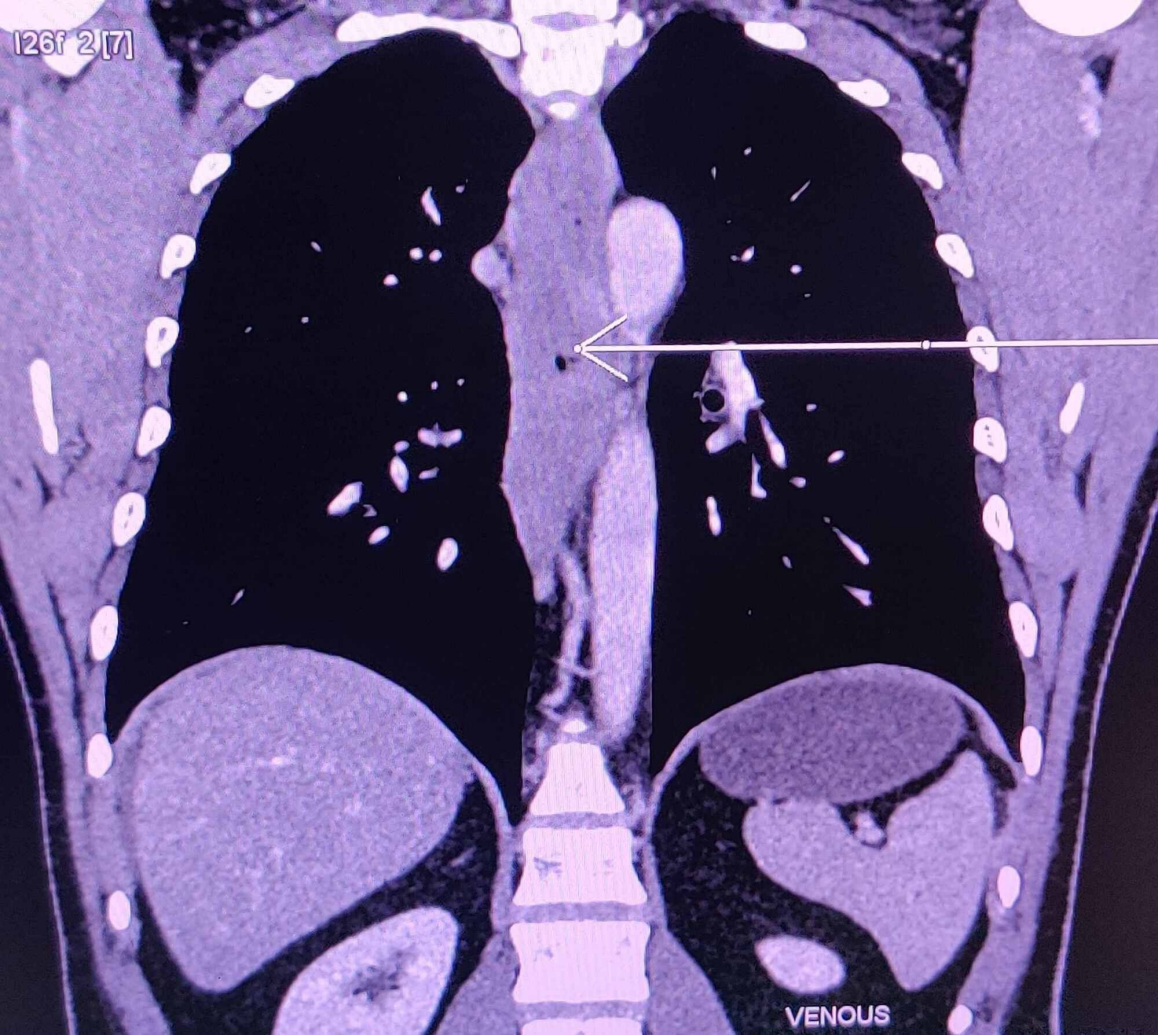
Contrast-enhanced computed tomography (CECT) of the abdomen showing circumferential wall thickening of esophagus (arrow)

A 44-year-old male with a history of bronchial asthma presented with gradually progressive abdominal distension for 10 days, associated with diffuse pain abdomen and one-two episodes of loose stool per day. He had a similar history of abdominal distension thrice in the last 15 years, empirically received antitubercular therapy and diuretics. On clinical examination, he had ascites with positive shifting dullness. On investigation, he was found to have low serum ascites albumin gradient (serum albumin 4.4 mg/dl, ascitic fluid albumin 3.4 mg/dl) with eosinophilic predominance (total cell 17854/mm^3^, mononuclear cell of 4%, polymorphonuclear cells of 96%, out of which eosinophils were 90%) with adenosine deaminase of 7.13 IU. Ascitic fluid tapping was done and cartridge-based nucleic acid amplification test (CBNAAT) of ascitic fluid was negative. Routine investigations of blood were within the normal range. A peripheral blood smear was showing eosinophilia with an absolute eosinophil count of 6750/mm^3^. Vitamin B_12_ level was 252 pg/ml, serum IgE of 1150 IU/ml, serum tryptase level was 1.23 mcg/L, amylase was 29 U/L, and lipase was 5 U/L, stool ovoparasite test was negative, and serology for hepatitis-B and hepatitis-C were non-reactive. Ultrasonography of the abdomen and pelvis showed no liver or renal disease except mild ascites. 2D echocardiography revealed no abnormality. Contrast-enhanced computed tomography (CECT) of the abdomen showed circumferential wall thickening of the esophagus (Figure 1).

**Figure 2:**
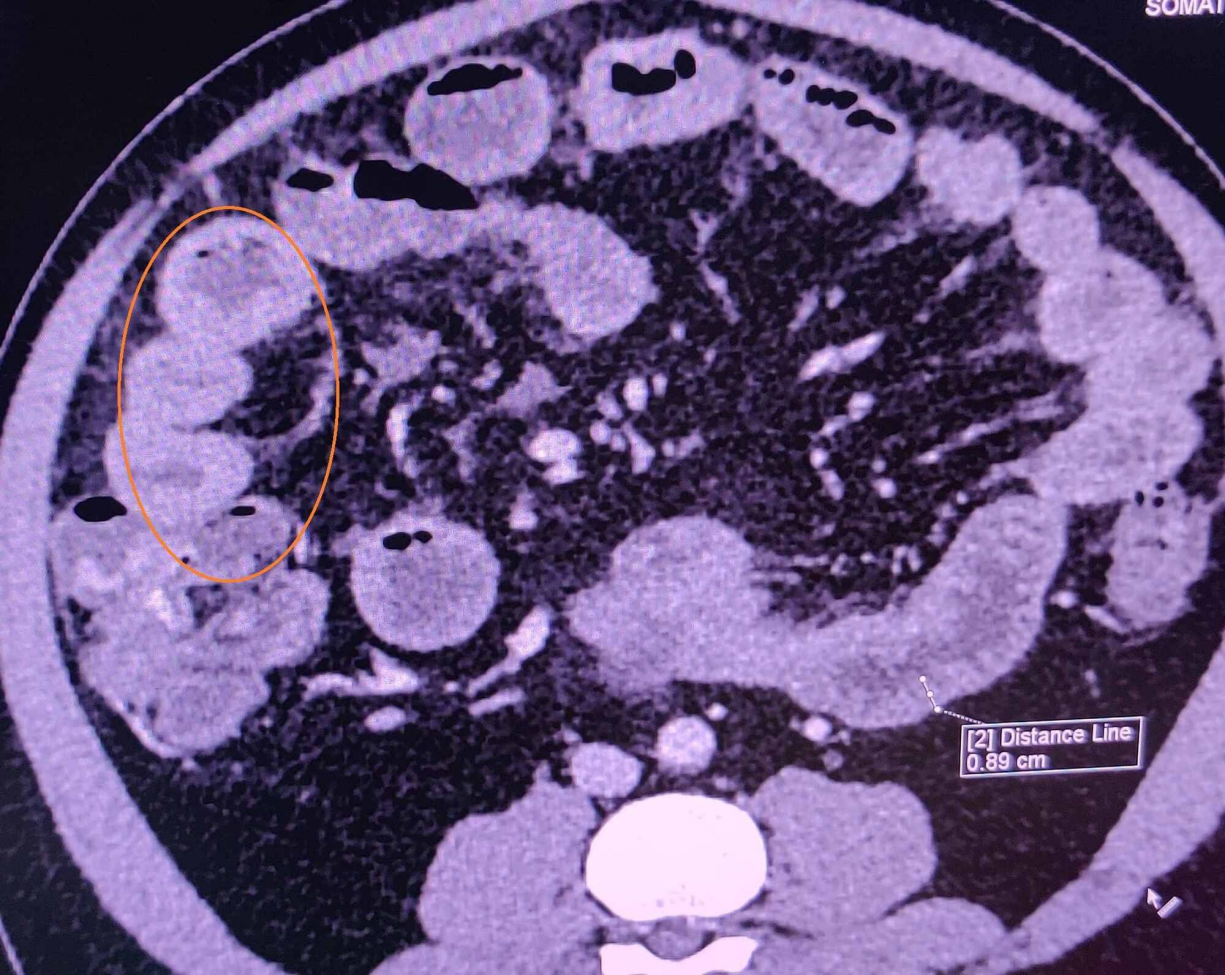
Contrast-enhanced computed tomography (CECT) of the abdomen showing wall thickening of ascending colon

It also showed mid to distal ileal loops and ascending colon (Figure 2).

Upper gastrointestinal endoscopy revealed a mucosal ringed esophagus with corporo antral erythema. Endoscopic biopsy from the esophagus showed esophagitis dessicans superficialis from the antrum, and the body showed Helicobacter pylori-associated chronic multifocal gastritis. Colonoscopy and colonoscopic biopsy were normal. Bone marrow biopsy revealed eosinophilia of 15%. The patient was managed with tablet prednisolone 20 mg twice daily for two weeks, then gradual tapering over one month. The patient responded to treatment with review ultrasonography of the abdomen and pelvis, showing no ascites after one week and serum eosinophil count became normal (3%).

## Discussion

Eosinophilic gastrointestinal disorder is a rare group of heterogeneous diseases consisting of eosinophilic esophagitis, eosinophilic gastroenteritis, and eosinophilic colitis and characterized by eosinophilic infiltration of gastrointestinal tract mucosa with subsequent inflammation, without any obvious cause of eosinophilia (e.g., drug reactions, parasitic infections, and malignancy) [1]. In 1937, it was first reported by Kaijser [2]. It is strongly associated with atopic disease with a family history of allergy in 3/4th of the cases [3]. EGID is more common in the third- to fifth-decade adult males [4]. After getting matured in the bone marrow and undergoing selective expansion, eosinophils remain in peripheral circulation for a short duration, and from there, they are trafficked to specific tissues like the gastrointestinal tract and interact with endothelium to manifest varied inflammation with the help of IL5, chemokines (eotaxin), platelet-activating factor, cysteinyl leukotriene C4 [5].

It can be of three subtypes: mucosal variety (most common 70%), presenting with diarrhea, melena, and iron deficiency anemia, protein-losing enteropathy; muscularis variety (20%) manifests as intestinal obstruction; and the least common, serosal variety (10%), shows peripheral eosinophilia and exudative ascites [6]. Rare presentations can be obstructive jaundice due to biliary tract involvement and extraintestinal manifestations like hepatitis, splenitis. Common differentials of eosinophilic ascites are parasitic infections (strongyloidiasis, Toxocara), abdominal tuberculosis, Churg Strauss vasculitis, malignancies (lymphoma, peritoneal metastasis), hypereosinophilic syndrome, chronic pancreatitis, etc. [7]. The diagnostic challenge about subserosal EGID is a rarity, nonspecific clinical presentation, non-diagnostic endoscopy since the biopsy sample is mostly taken from the mucosa. Diagnostic criteria include the presence of gastrointestinal symptoms, no evidence of parasitic or extraintestinal manifestations, and gastrointestinal tract biopsy showing eosinophilic infiltration, or radiological findings characteristics of disease with peripheral eosinophilia or eosinophilic ascites [3].

Eosinophilic esophagitis on upper gastrointestinal endoscopy might show longitudinal furrowing, mucosal rings, strictures, ulceration, and polyps, whereas in biopsy showing > 20 to 24 eosinophils per highpower field is diagnostic [8, 9]. On the other side, on colonoscopy, eosinophilic colitis reveals patchy erythema, loss of vascularity and lymphonodular hyperplasia, and biopsy shows focal eosinophilic aggregates in lamina propria, crypt epithelium with or without multinucleated giant cells. Small bowel biopsy in eosinophilic gastroenteritis, there is extracellular deposition of eosinophilic granules like major basic protein, eosinophilic cationic protein.

Treatment of EGID consists of oral steroids (prednisolone 20-40 mg/day in divided doses) for two weeks, followed by tapering over the next two weeks with excellent response [10]. Alternate second-line drugs include mast cell stabilizers (sodium cromoglycate, ketotifen), leukotriene receptor antagonist (montelukast), an anti-IgE monoclonal antibody (omalizumab), anti-IL 5 monoclonal antibody (mepolizumab). Complete remission of symptoms occurs in 80% of cases within one week and normalization of eosinophil count within two weeks. There is less chance of recurrence; however, it responds well with short course steroids if it recurs. In steroid-resistant cases, immunosuppressants like azathioprine, cyclosporine can be tried [11].

## Conclusions

In cases of eosinophilic ascites, abdominal pain, ascites, and peripheral hypereosinophilia in the absence of characteristic upper gastrointestinal endoscopic biopsy findings don't rule out the disease. Awareness of the condition, timely diagnosis, and early treatment with oral steroids carry excellent responses. A multidisciplinary team comprising of a physician, gastroenterologist, nutrition specialist might be a better approach to deal with these types of cases.
